# Draft Genome Sequence of the Root-Colonizing Fungus *Trichoderma harzianum* B97

**DOI:** 10.1128/genomeA.00137-17

**Published:** 2017-03-30

**Authors:** Stéphane Compant, Jonathan Gerbore, Livio Antonielli, Aline Brutel, Monika Schmoll

**Affiliations:** a AIT Austrian Institute of Technology, Center for Health and Bioresources, Tulln, Austria; b Biovitis, Saint-Étienne-de-Chomeil, France

## Abstract

*Trichoderma harzianum* is one of the most beneficial microorganisms applied on diverse crops against biotic and abiotic stresses and acts also as a plant growth-promoting fungus. Here, we report the genome of *T. harzianum* B97, originating from a French agricultural soil and used as a biofertilizer that can tolerate abiotic stresses.

## GENOME ANNOUNCEMENT

*Trichoderma* spp. are well known for their applications against different kinds of diseases and stresses, as well as for stimulating plant growth. They can produce antibiotics against a wide range of pathogens, parasitize pathogenic fungi, induce systemic resistance in plants, reduce abiotic stresses, and colonize plants, making them adequate microorganisms for commercial application (1, 2).

*Trichoderma harzianum* strain B97 was isolated by Biovitis from agricultural soil in France and investigated further due to its efficient stimulation of plant growth. It has been studied for many years as a biofertilizer on wheat and maize and it was found to solubilize phosphate as well as alleviate abiotic stresses. These results encouraged us to produce a complete sequence of *T. harzianum* B97.

*T. harzianum* B97 was sequenced using Illumina 2 × 125-bp paired-end sequencing technology (1.04 Gb total, average coverage 25.4 ± 18.5×) and single nucleotide polymorphism (SNP) analysis was performed at GATC Biotech AG, Konstanz, Germany. Reads were assembled using SPAdes version 3.8.1 (3). The draft genome of *T. harzianum* B97 consists of 1,054 scaffolds (≥0 bp), with 951 scaffolds larger than 1,000 bp. The total assembly length was 40.68 Mb, and 11,961 predicted protein-coding genes were detected. The quality of the genome assembly was evaluated using QUAST version 4.3 (4) and Qualimap version 2.2 (5). Blobtools were used to ascertain the unintended presence of contaminants (6). A single-copy ortholog analysis was performed with BUSCO, which reported a genome completeness of 99% (7). An unsupervised protein-coding gene search was assessed using GeneMark-ES optimized for fungi (8); 11,961 genes were determined, of which 91% showed a similarity between 97 and 100% when a conditional reciprocal best BLAST search (9) was applied against *T. harzianum* strain CBS226.95 proteins. In total, the sequence analysis revealed 148,033 SNPs in the sequence of *T. harzianum* B97 when compared to the sequenced *T. harzianum* strain CBS226.95 version 1.0 (http://genome.jgi.doe.gov/Triha1/Triha1.home.html). Of those, 67,774 SNPs were downstream of open reading frames (ORFs), 12,389 were upstream of ORFs, and 10,499 represent synonymous mutations that did not cause alterations in the encoded protein sequence compared to the reference strain.

However, 12,387 SNPs that caused alterations in the encoded proteins (nonsynonymous SNPs) were also detected. Notably, 21 genes lost the start codon due to the alteration in the genome and are now likely to be nonfunctional. Moreover, 517 genes gained a stop codon resulting in premature termination of the translation, which will hence influence or abolish the function of the encoded protein.

Functional analysis of genes containing nonsynonymous SNPs compared to the sequenced strain (JGI) revealed a strong and significant enrichment of genes involved in metabolism (*P* value = 0.0), particularly amino acid metabolism (*P* value = 1.57e−03), and also of other diverse compounds, including those involved in secondary metabolism (*P* value = 7.46 e−03). Interestingly, genes involved in DNA repair were also significantly enriched (*P* value = 3.37e−03).

Availability of the genome sequence of *T. harzianum* B97 will contribute to the investigation into the relevance of different genotypes for phenotypes that are important for efficient alleviation of abiotic stresses and stimulation of plant growth in agriculture.

### Accession number(s).

The genome sequence of *T. harzianum* B97 has been deposited in GenBank under the accession number MRYK00000000 (BioProject PRJNA357189). The version described in this paper is the first version, MRYK00000000.1.
