# Correction for Compant et al., “Draft Genome Sequence of the Root-Colonizing Fungus *Trichoderma harzianum* B97”

**DOI:** 10.1128/genomeA.00549-17

**Published:** 2017-06-08

**Authors:** Stéphane Compant, Jonathan Gerbore, Livio Antonielli, Aline Brutel, Monika Schmoll

**Affiliations:** a AIT Austrian Institute of Technology, Center for Health and Bioresources, Tulln, Austria; b Biovitis, Saint-Étienne-de-Chomeil, France

## AUTHOR CORRECTION

Volume 5, no. 13, e00137-17, 2017, https://doi.org/10.1128/genomeA.00137-17. Page 2: The Acknowledgments should include the following. “We thank Christian Kubicek, Irina Druzhinina, and Igor Grigoriev for providing access to unpublished genome data produced by the U.S. Department of Energy Joint Genome Institute, a DOE Office of Science User Facility, supported by the Office of Science of the U.S. Department of Energy under contract no. DE-AC02-05CH11231.”

